# Validation of the Use of Dried Blood Samples for the Detection of *Toxoplasma gondii* Antibodies in Stray Cats (*Felis s. catus*)

**DOI:** 10.3390/pathogens10070864

**Published:** 2021-07-08

**Authors:** Julie Alice Simon, Dominique Aubert, Régine Geers, Isabelle Villena, Marie-Lazarine Poulle

**Affiliations:** 1 Epidémio-Surveillance et Circulation des Parasites dans les Environnements (ESCAPE), EA 7510, CAP SANTE, Université de Reims Champagne Ardenne, CEDEX, 51095 Reims, France; julie.rabeisensimon@gmail.com (J.A.S.); daubert@chu-reims.fr (D.A.); rgeers@chu-reims.fr (R.G.); ivillena@chu-reims.fr (I.V.); 2 Centre de Recherche et de Formation en Eco-éthologie (CERFE), Université de Reims Champagne-Ardenne, 08240 Boult-aux-Bois, France; 3 Laboratoire de Parasitologie-Mycologie, Centre National de Référence de la Toxoplasmose, Centre de Ressources Biologiques *Toxoplasma*, CHU Reims, CEDEX, 51092 Reims, France

**Keywords:** toxoplasma serology, field sampling, sensitivity, specificity, concordance, kappa coefficient

## Abstract

If validated beforehand, the analysis of dried blood on blotting paper (BP samples) is very useful for monitoring free-ranging animals. We aimed to validate this method for the detection of antibodies against *Toxoplasma gondii* in stray cats. We used the modified agglutination test (MAT) in 199 sample pairs of sera and BP samples from 54, 39, 56, and 50 cats trapped during four periods in five dairy farms. Screening was at 1:6, 1:12, and 1:24 dilutions. The cut-off value was at MAT titre ≥ 24, but MAT titre ≥ 12 was also considered for BP samples that often have a higher dilution level. Depending on the period, sample type, and cut-off value, sensitivity of the analysis of the BP sample vs. serum varied from 87.1% to 100% and specificity ranged from 72.22% to 100%. The concordance values and Kappa coefficient showed a substantial to excellent agreement between the results of the two methods, whatever the cut-off value. These findings quantifiably validate the use of MAT on BP samples for the detection of antibodies to *T. gondii* in stray cats, but we recommend expressing results from BP samples with several cut-off values as the MAT titres tend to be lower than those of sera.

## 1. Introduction

The collection and storage of blood samples on filter paper (BP samples) has been common in human medicine for 50 years [1] and has been increasingly used in the last two decades to diagnose infectious diseases and study the circulation of pathogens in wildlife [2,3,4], dogs [5,6], and livestock [7]. It consists of taking about 50 µL of blood on strips or spots of an absorbent paper (filter or blotting paper) and letting the sample dry for a few minutes before storing it at room temperature until the test [8]. Therefore, compared to the traditional blood collection in glass tubes, the use of BP samples is more cost-efficient and easy to collect in the field, as it does not require drawing blood with a needle and syringe, centrifuging the samples and storing the sera at 4 °C. This method also has the advantage of allowing sampling by untrained staffs, such as hunters collecting a few drops of blood from freshly killed animals [9,10], and at short time-intervals on live animals, as required for the study of the temporal dynamics of exposure to pathogens [11]. However, results from the analysis of BP samples must be validated and calibrated by comparison with a reference method before being used to assess the serological status of a given species in relation to a given infection [12,13].

Toxoplasmosis is one of the most common zoonotic infections worldwide. The causative agent is *Toxoplasma gondii*, a protozoan parasite that naturally circulates between felids (definitive hosts), the environment, and warm-blooded species (intermediate hosts). The vast majority of estimates of the prevalence of *T. gondii* antibodies in animal populations are based on the analysis of sera, considered as the “gold standard” method (see review in [14]), but BP samples have already been used to detect antibodies in wild rodents [15,16,17], wild and domestic birds [18,19], carnivores [20,21,22], and ungulates [7,23]. However, with the notable exception of the study conducted by Sharma et al. [22], validation of the use of BP samples to assess the serological status of wildlife species naturally infected by *T. gondii* have rarely been done. Nogami et al. [24] showed that it was possible to detect antibodies to *T. gondii* in experimentally infected domestic cats (*Felis s. catus*) from blood eluates dried on absorbent paper and stored for several months. To our knowledge, only Simon et al. [11] and Bolais et al. [25] have used this technique to detect antibodies to *T. gondii* in cats naturally exposed to this parasite. Bolais et al. [25] found identical results of IgG antibody detection in cats using the modified agglutination test (MAT) on serum and dried blood samples. Their 36 positive samples showed high titres for both methods, except for three cases that showed discordance between titres obtained with MAT on serum or dried blood spots. The aim of the study was to confirm the relevance of using MAT on BP samples to detect *T. gondii* antibodies in cats by comparing the MAT results from BP and serum samples using numerical estimators.

## 2. Results

Either based on results from BP or serum samples, the prevalence of antibodies to *T. gondii* in the stray cat population varied from about 60% to 80% depending on the period and the cut-off value (Table 1). With MAT titre ≥24, the number of positive BP samples was equal to the number of positive sera in periods 3 and 4 (34 and 36 positive samples respectively, Table 1) and was lower than the number of positive sera in period 1 (34 versus 36) and period 2 (27 versus 31). With MAT titre ≥12 for BP samples, the number of positive BP samples was higher than the number of positive sera in period 1 (41 versus 36), period 3 (35 versus 34) and period 4 (37 versus 36), but results from BP and serum samples matched in period 2 (31 positive samples in both cases, Table 1).

Sensitivity and specificity were, respectively, >87% and >92%, for all trapping periods (Table 2). Positive predictive values ranged from 97.06% to 100% and from 87.80% to 100% across periods with a titre of MAT ≥24 and ≥12 as respective cut-off values (Table 2). Negative predictive values ranged from 66.67% to 100% across periods, and were equal to 100% for all periods with a titre of MAT ≥24 and ≥12 as respective cut-off values (Table 2). Concordance values ranged from 89.74% to 100% and from 90.74% to 100% with titres of MAT ≥24 and ≥12 as respective cut-off values (Table 2). The Kappa coefficient ranged from 0.73 to 1.00 (Table 2) showing a substantial to excellent agreement between the results obtained from the two types of samples.

**Figure 1:**
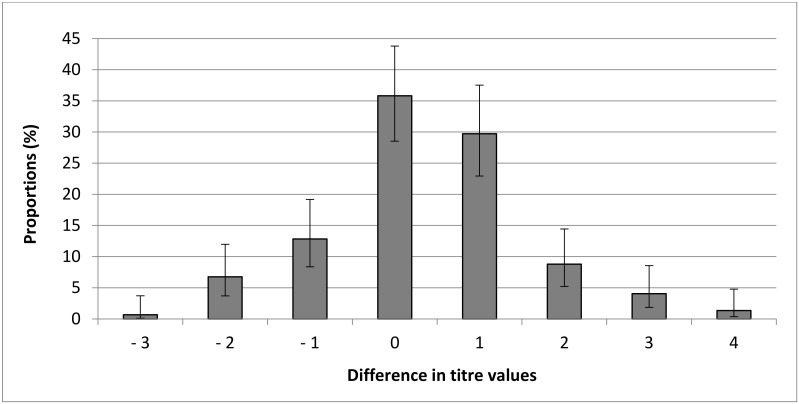
Distribution of differences in modified agglutination test (MAT) titre between blood dried on blotting paper and serum for each pair of cat samples.

Finally, 65 of the 148 paired samples having a MAT titre >0 had lower MAT titres from BP than serum samples (43.92% [95% CI: 36.18–51.97]) and 44/148 (29.73% [95% CI: 22.95–37.53]) of samples had only one titre of difference. However, 35.81% [95% CI: 28.53–43.80] had the same MAT titre between BP samples and the serum sample, and 20.27% [95% CI: 14.58–27.46] of samples had higher MAT titres from BP than serum samples (Figure 1).

**Figure 2:**
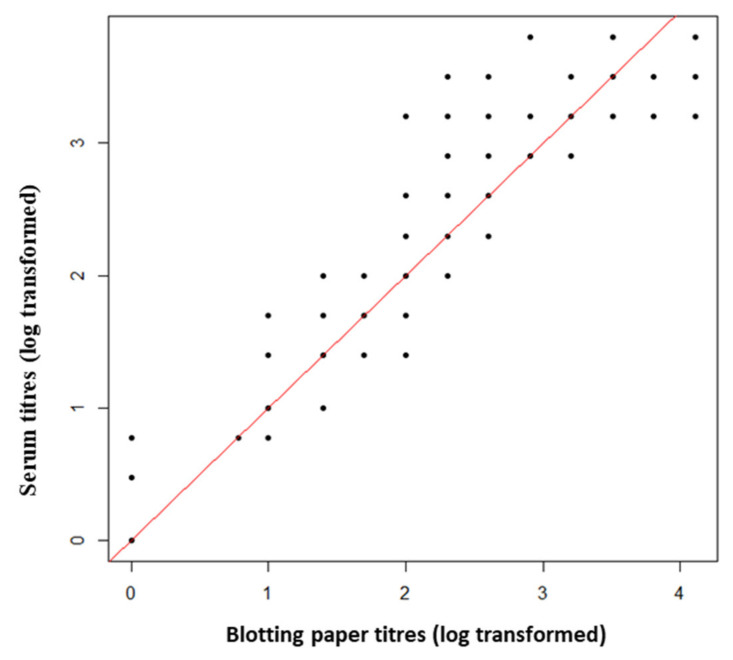
Comparison of modified agglutination test (MAT) titres obtained from dry blood stored on blotting paper and serum for each pair of cat samples. The red line represents the equality of titres between the two types of samples.

Dried blood stored on blotting paper (i.e., BP samples) tended to obtain lower MAT titres than serum samples whatever the titre value (Figure 2).

## 3. Discussion

We carried out this study on five populations of stray cats naturally exposed to *T. gondii* in their rural environment and estimated the immune status of cats with respect to this parasite by testing their sera with MAT, recognized as a sensitive and specific test for the detection of antibodies against *T. gondii* in cat sera [26,27,28]. Astles [29] considered that a method is reliable when its sensitivity and specificity are greater than or equal to 80%. Therefore, the sensitivity, specificity, and predictive values we assessed in this study indicate that using MAT on BP samples is reliable in identifying the presence of antibodies to *T. gondii* in domestic cats. Furthermore, according to the concordance and Kappa coefficient values, MAT results from BP samples well match those from sera. In accordance with results from the dog survey conducted by Holton et al. [30], the Kappa coefficient values indicate a “substantial” matching (values between 0.61 and 0.80) to “almost perfect” matching (values between 0.81 and 0.99). However, results from BP samples were generally one MAT titre lower than from serum samples. This finding is in accordance with results from BP samples used to detect others’ antibodies or circulating metabolites [31,32]. The elution step that is required to dissolve the BP sample may explain the lower titres obtained when compared to standard serum samples [31]. These lower titres can result in a misclassification in the assignment of serological status, notably producing false-negative results for seropositive individual showing an agglutination reaction at titres <24. Lowering the cut-off value used to discriminate between seropositive and seronegative MAT results by one titre could correct this bias (see [31], for example). However, we observed that it sometimes results in a specificity decrease. The presentation of a range of cut-off values could be a good alternative, as recommended for other screening methods and for other pathogens, such as anthrax [33]. The present study also evidenced variations in the estimates of sensitivity, specificity, and other parameters between sampling periods. This may be partly explained by differences in cats sampled at each period and/or by MAT titre variations from one period to another for the same sampled cat. In conclusion, our findings validate the use of BP samples for the MAT detection of anti-*Toxoplasma gondii* antibodies as a good alternative to the conventional method of blood collection and serological testing of sera. However, as the MAT titres obtained tend to be lower with BP samples, we recommend expressing results with several cut-off values in order to reduce errors in estimating prevalence and incidences, which are key parameters in eco-epidemiology. Some other factors should also be tested to optimise the use of BP samples, such as the quality of the absorbent paper [23], the quantity of dried blood used [34], and the duration and condition of storage of BP samples [35]. In addition, the collection of several blood samples from the same individual in a single collection session would allow the replicability of the method, as usual in serological test validation processes [12].

## 4. Materials and Methods

### 4.1. Sampling

The present study is complementary to that of Simon et al. [11] who conducted cat-trapping on five dairy farms in France in April 2015, July 2015, October 2015, and January 2016. These periods respectively led to the sampling of 54, 39, 56, and 50 domestic cats. All were free-roaming cats that relied on predation for survival and were not under veterinary care (i.e., stray cats). Cats were captured in baited cage traps, anaesthetised, and individually identified with subcutaneous passive integrated transponder (PIT) tags (see [11] for more details of the procedure). On each capture, punctures on the marginal ear vein using a sterile needle allowed us to collect two to four drops of blood to soak a 2 to 3 cm^2^ surface area of blotting paper (BP, Whatman 3MM CHR) bearing the cat’s ID number. The remaining collected blood (≤1 mL) was stored in glass tubes and centrifuged to obtain serum. Blotting paper storage began with overnight at room temperature in the individual compartment of a closed drying box and continued in an individual envelope labelled with the cat’s ID and the collection date, when the blood was completely dry. BP and serum sampled with the same information reported were stored at −20 °C and analysed within a month. Cats were tested only once per trapping period, but some were tested over two or three trapping periods.

### 4.2. Serological Examination

We cut out a 1 cm × 1 cm strip of BP using clean scissors. We eluted dry blood on a BP patch in 300 μL of phosphate-buffered saline solution (PBS, pH 7.2), and incubated the eluate under agitation overnight before centrifugation and testing for the presence of IgG antibodies to *T. gondii* using the modified agglutination test (MAT) [36]. The same person (RG) performed all the serological analyses to avoid bias in agglutination detection. MAT antigen consisted of formalinized tachyzoïtes produced at the Laboratory of Parasitology, National Centre on Toxoplasmosis, Reims, France. Sera and eluates were first screened using 1:6, 1:12, and 1:24 dilutions in phosphate-buffered saline solution (PBS, pH 7.2). Those agglutinating the antigen at one (or more) of these screening dilutions were further tested in a serial two-fold dilution, to a maximum dilution of 1:12800. Serum samples with agglutination at MAT titre ≥24 were considered positive for the presence of *T. gondii* antibodies [26,27,37]. However, as the level of dilution of the eluate obtained after BP elution is generally not known, the dilution level is often higher in eluates than in sera and may result in lower MAT titres [31,32]. For this reason, we used two cut-off values for eluates: MAT titre ≥24 and MAT titre ≥12.

### 4.3. Analysis of the Results

We paired results obtained with BP and sera samples collected on the same cat at the same moment. We calculated the sensitivity and specificity of the BP method as, respectively, the proportion of positive results for both methods relative to the total number of positive results and the proportion of negative results for both methods relative to the total number of negative results. The number of positive results obtained with sera on the number of positive results obtained with BP samples (positive predictive value) and the number of negative results from sera on the number of negative results from BP samples (negative predictive value) were also calculated, as well as the concordance and Kappa coefficients [38]. All these parameters were expressed with their Wilson 95% confidence intervals as recommended for the small sample size [39]. Kappa values ≤0.40 represent slight to poor agreement, those between 0.40 and 0.60 represent moderate to good agreement, those between 0.61 and 0.80 represent substantial agreement, and those ≥0.81 represent excellent agreement [38]. Analysis of the differences in titres between each pair of samples allowed a quantitative comparison of results obtained with BP and serum samples.

## Figures and Tables

**Table 1:** The serological prevalence of *Toxoplasma gondii* assessed from results of the modified agglutination test (MAT) on sera and dried blood on blotting paper in the stray cats trapped on five dairy farms in France during four periods.

Trapping Period	Positive Serum Samples with MAT Titre ≥24	Positive BP Samples with MAT Titre Toxoplasma ≥24	Positive BP Samples with MAT Titre ≥12
Period 1	36/54 (66.67%) [95% CI: 53.36–77.76]	34/54 (62.96%) [95% CI: 49.63–74.58]	41/54 (75.92%) [95% CI: 63.05–85.36]
Period 2	31/39 (79.49%) [95% CI: 64.47–89.22]	27/39 (69.23%) [95% CI: 53.58–81.43]	31/39 (79.49%) [95% CI: 64.47–89.22]
Period 3	34/56 (60.71%) [95% CI: 47.63–72.42]	34/56 (60.71%) [95% CI: 47.63–72.42]	35/56 (62.50%) [95% CI: 49.41–73.99]
Period 4	36/50 (72%) [95% CI: 58.33–82.53]	36/50 (72%) [95% CI: 58.33–82.53]	37/50 (74%) [95% CI: 60.45–84.13]

**Table 2:** Comparison of results of the modified agglutination test to detect *Toxoplasma gondii* antibodies from blood samples stored on blotting paper vs. serum samples in the stray cats trapped on five dairy farms in France during four periods.

MAT Titre ≥24					
	Period 1	Period 2	Period 3	Period 4	Mean
Se	91.67 [78.17–97.13]	87.10 [71.15–94.87]	100 [89.85–100]	100 [90.36–100]	94.69 ± 0.06
Sp	94.44 [74.24–99.01]	100 [67.56–100]	100 [85.13–100]	100 [78.47–100]	98.61 ± 2.78
PPV	97.06 [85.08–99.48]	100 [87.54–100]	100 [89.85–100]	100 [90.36–100]	99.26 ± 1.47
NPV	85.00 [63.96–94.76]	66.67 [39.08–86.19]	100 [85.13–100]	100 [78.47–100]	87.92 ± 15.83
Co	92.59 [82.45–97.08]	89.74 [76.42–95.94]	100 [93.58–100]	100 [92.87–100]	95.58 ± 5.23
Kappa	0.84 [0.68–0.99]	0.73 [0.49–0.98]	1	1	0.89 ± 0.13
**MAT Titre ≥12**					
	**Period** **1**	**Period** **2**	**Period** **3**	**Period** **4**	**Mean**
Se	100 [90.36–100]	100 [88.97–100]	100 [89.85–100]	100 [90.36–100]	100.00 ± 0.00
Sp	72.22 [49.13–87.50]	100 [67.56–100]	95.45 [78.20–99.19]	92.86 [68.53–98.73]	90.13 ± 12.30
VPP	87.80 [74.46–94.68]	100 [88.97–100]	97.14 [85.47–99.49]	97.30 [86.18–99.52]	95.56 ± 5.33
VPN	100 [77.19–100]	100 [67.56–100]	100 [84.54–100]	100 [77.19–100]	100.00 ± 0.00
Co	90.74 [80.09–95.98]	100 [91.03–100]	98.21 [90.55–99.68]	98 [89.5–99.65]	96.79 ± 4.12
Kappa	0.78 [0.59–0.96]	1	0.96 [0.89–1.03]	0.95 [0.85–1.05]	0.92 ± 0.10

MAT = modified agglutination test; Se = Sensitivity; Sp = specificity, PPV = positive predictive values; NPV = negative predictive values, Co = concordance coefficient; Kappa = Kappa coefficient; BP samples = dried blood stored on blotting paper; MAT = modified agglutination test (MAT). In square brackets: Wilson 95% confidence intervals.

## Data Availability

The data presented in this study are available in supplementary material (Table S1).

## Supplementary Materials

The following are available online at https://www.mdpi.com/article/10.3390/pathogens10070864/s1, Table S1: Titers of modified agglutination tests conducted on paired samples of dried blood and sera from stray cats captured on five French dairy farms during four consecutive periods in 2015–2016.
